# Proteomic Biomarkers of Retinal Inflammation in Diabetic Retinopathy

**DOI:** 10.3390/ijms20194755

**Published:** 2019-09-25

**Authors:** Hannah Youngblood, Rebekah Robinson, Ashok Sharma, Shruti Sharma

**Affiliations:** 1 Department of Cellular Biology and Anatomy, Augusta University, Augusta, GA 30912, USA; 2 Medical College of Georgia, Augusta University, Augusta, GA 30912, USA; 3 Center for Biotechnology and Genomic Medicine, Augusta University, Augusta, GA 30912, USA; 4 Department of Population Health Sciences, Augusta University, Augusta, GA 30912, USA; 5 Culver Vision Discovery Institute, Augusta University, Augusta, GA 30912, USA; 6 Department of Ophthalmology, Augusta University, Augusta, GA 30912, USA

**Keywords:** Diabetic retinopathy, Retinal inflammation, Proteomics, Biomarkers

## Abstract

Diabetic retinopathy (DR), a sight-threatening neurovasculopathy, is the leading cause of irreversible blindness in the developed world. DR arises as the result of prolonged hyperglycemia and is characterized by leaky retinal vasculature, retinal ischemia, retinal inflammation, angiogenesis, and neovascularization. The number of DR patients is growing with an increase in the elderly population, and therapeutic approaches are limited, therefore, new therapies to prevent retinal injury and enhance repair are a critical unmet need. Besides vascular endothelial growth factor (VEGF)-induced vascular proliferation, several other mechanisms are important in the pathogenesis of diabetic retinopathy, including vascular inflammation. Thus, combining anti-VEGF therapy with other new therapies targeting these pathophysiological pathways of DR may further optimize treatment outcomes. Technological advancements have allowed for high-throughput proteomic studies examining biofluids such as aqueous humor, vitreous humor, tear, and serum. Many DR biomarkers have been identified, especially proteins involved in retinal inflammatory processes. This review attempts to summarize the proteomic biomarkers of DR-associated retinal inflammation identified over the last several years.

## 1. Introduction

Diabetic retinopathy (DR) is the leading cause of irreversible blindness in the developed world and contributes to the majority of blindness in working-age adults, affecting more than four million individuals [1,2,3]. The prevalence of this sight-threatening neurovasculopathy is expected to increase with longer life expectancies and the growing elderly population [1,4,5]. DR is the most common microvascular complication arising from diabetes, and diabetic patients have a twenty-five-fold greater risk of blindness than non-diabetic individuals. Since DR arises as a result of extended exposure to hyperglycemia, it occurs in almost all type I diabetics and the majority of type II diabetics, thereby nearly tripling their treatment costs [6,7,8,9].

DR is characterized by leaky retinal vasculature, retinal ischemia, angiogenesis, and retinal inflammation. These pathologies manifest clinically as cotton-wool spots, exudates, small tortuous veins, aneurysms, and areas of hemorrhage, which may result in reduced acuity, loss of color sensitivity, and problematic night vision [1,10,11]. While the underlying cause of DR is prolonged hyperglycemia, still much is unknown about the precise pathogenesis of the disease [12,13]. Inflammation triggered by increased glycolytic metabolites has been well established in individuals with DR, and retinal inflammation leads to vascular permeabilization and loss of the blood-retinal barrier [1,5,13,14,15,16,17,18,19]. As a result, macular edema (diabetic macular edema (DME)) may develop, reducing central vision [1,5,14,15,16,17]. Retinal ischemia may also induce the growth of new, small, abnormal blood vessels into the central portion of the posterior segment, a condition known as proliferative diabetic retinopathy (PDR) [1,5,10,11,16,17]. As these vessels grow and attach to the surface of the vitreous body, they easily rupture and may cause retinal detachment, leading to vision loss [1,16,17,20]. These two forms of DR may occur separately or concurrently [21,22].

Diagnosis of DR is made based on clinical features observed during comprehensive eye examination [23]. Current recommendations include initial screening exams for type I diabetic patients three to five years after disease onset and, for type II diabetic patients, at the time of diagnosis, with regular follow-up with an ophthalmologist experienced in treating DR [23]. Risk factors for DR include the duration of diabetes as well as modifiable risk factors such as poor glycemic control, hypertension, dyslipidemia, and obesity [24,25,26,27,28]. Managing blood glucose, lipid, and A1c levels are the primary means of reducing the risk of developing DR among diabetic patients, and have been shown to reduce severity upon its incidence [29,30,31].

Currently, treatment strategies for DR are limited to more advanced stages of the disease when retinal damage becomes symptomatic [11]. Standard of care for most patients includes intravitreal injections of corticosteroids or anti-vascular endothelial growth factor (VEGF) agents, such as bevacizumab, ranibizumab, pegaptanib, or aflibercept, with therapeutic efficacy dependent on a patients’ baseline visual acuity [2,3,5,11,15,32,33,34,35,36,37,38,39,40]. In advanced cases, laser photocoagulation may be used to reduce the amount of blood vessels entering the posterior segment of the eye [2,39]. While laser photocoagulation does control the effects of neovascularization and some macular edema, it typically achieves its effect through destructive processes. Anti-VEGF therapy is far less destructive, but is primarily aimed at limiting disease progression through repeated intra-ocular injections, which are not without adverse effects [35,40,41,42,43,44,45,46,47,48,49,50,51,52,53,54,55]. Therefore, new therapies to prevent retinal injury and enhance repair remain a critical unmet need.

In diabetes, elevated blood glucose levels alter critical homeostatic mechanisms, resulting in changes to the proteomic microenvironment crucial to proper cellular function. Several of these changes, including altered expression of inflammatory mediators and leukocyte adhesion molecules, have been correlated to the progression of DR. A better understanding of the precise proteomic changes that occur during DR development and progression could provide new insight into disease pathophysiology and may lead to the development of novel treatment options.

## 2. Use of Proteomics Technologies for Biomarker Discovery in Diabetic Retinopathy

The last several decades have seen a rapid advancement in methodology, engineering, and equipment design, and it is now possible to generate large amounts of data using small amount of samples. Remarkable advances in mass spectrometry (MS) techniques are allowing for deeper analysis and the identification of more proteins at faster speeds [7,22,56]. In addition, MS technology is useful for identification of post-translational modifications and variants of proteins [16,22,57,58,59,60]. The use of high throughput proteomics has expanded to include ophthalmic investigations. Numerous studies have been conducted on samples from DR patients including tear, cornea, aqueous humor (AH), lens, vitreous humor (VH), retina, and serum [61] using proteomic approaches such as two-dimensional difference gel electrophoresis (2D-DIGE) coupled with MS [10,20,39,62,63,64,65], SDS-PAGE coupled with MS [16,66], liquid chromatography coupled with tandem MS (LC-MS/MS) [3,4,13,15,17,33,35,56,67,68,69,70,71,72], and bead-based multiplex immunoassays [73,74]. Several well-characterized biomarkers of DR have been identified, including complement component C3, intercellular adhesion molecule 1 (ICAM-1), interleukin-6 (IL-6), serum amyloid A protein (SAA), vascular endothelial growth factor (VEGF), etc. [4,16,66,72,75,76,77,78,79,80,81], as shown in Table 1.

## 3. Proteomic Changes in Biofluids Associated with Diabetic Retinopathy

**Figure 1:**
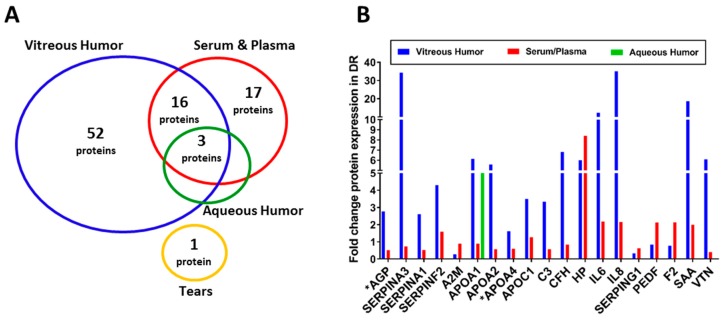
Summary of differentially expressed proteins identified in biofluids of DR patients. (**A**) The number of potential biomarkers of retinal inflammation in each biofluid, as identified by the studies discussed in this review. (**B**) The bar graphs represent fold change in expression of 19 biomarkers identified in at least two biofluids. * AGP and APOA4 proteins were detected in the AH of DR patients, but fold change was not available.

Increased vascular permeability and elevated levels of inflammatory proteins in serum and ocular biofluids (VH, AH, and tear) are the primary characteristics of retinal inflammation associated with DR [11,15,95,96,97,98,99,100,101]. While AH and tear are distally located and interact indirectly with the retina, both serum and VH have direct contact with the retina. On the other hand, AH and VH sample collection requires highly invasive procedures, whereas serum and tear samples can be collected using relatively non-invasive methods. The circulatory proteins in these biofluids can not only provide clues about DR pathogenesis, but can also serve as biomarkers, with diagnostic or prognostic potential [102,103,104]. Furthermore, proteomic changes in biofluids could offer a means of more personalized medicine. For example, plasma kallikrein, a central component of the pro-inflammatory kallikrein kinin system, has been shown to be elevated in the VH of DR patients [15]. The kallikrein kinin system works independently of VEGF regulation, and standard anti-VEGF intravitreal injection might not be effective in these subjects [11,15]. Several studies examining proteomic changes associated with DR using biofluids are discussed below and are summarized in Figure 1.

### 3.1. Serum

Diabetes is a systemic disease therefore, biomarkers found in circulation may not only be indicators of the disease, but also of the progression of specific diabetic complications. As DR is primarily a microvascular complication, proteomic changes involved in DR pathology may be evident in the systemic circulation. Serum is one of the most accessible and easily obtained biofluids, allowing for sample collection from both DR patients and healthy controls. The ease of sample collection relative to other relevant fluids like VH or AH provides sufficient power to detect potential proteomic differences in patients with various clinical stages of DR including non-proliferative DR (NPDR), PDR, and DME [105].

A number of serum proteins have been found to be altered in DR, including α-2-antiplasmin (SERPINF2), C-reactive protein (CRP), C-C motif chemokine 5 (CCL5), intercellular adhesion molecule 1 (ICAM-1), interleukin-6 (IL-6), pentraxin-related protein 3 (PTX3), pigment epithelium-derived factor (PEDF), plasminogen activator inhibitor 1 (PAI-1), serum amyloid A (SAA), soluble endothelial molecule-1 (sE-selectin), stromal cell-derived factor 1α (CXCL12), tumor necrosis factor α (TNF-α), vascular adhesion molecule 1 (VCAM-1), and vascular endothelial growth factor (VEGF) [84,85,86,87,88,90,91,92,93,94,105]. VEGF is one of the most well-established biomarkers associated with DR [87,92,93,94,106]. Its expression is promoted by retinal ischemia stemming from dyslipidemia and leukostasis and results in the initiation of neovascularization [105]. In our previous study, we identified five inflammatory proteins with significantly higher levels in the serum of type I diabetic patients with DR as compared to those without DR [74]. These proteins included C-responsive protein (CRP), ICAM-1, soluble glycoprotein 130 (sgp130), TNF receptor I, and VCAM-1 [74]. High levels of any of these five proteins significantly increased the odds of developing DR [74].

In another study, the inflammation-regulating proteins α2-HS-glycoprotein (AHSG), α1-acid glycoprotein (AGP), apolipoprotein A-1 (APOA1), and haptoglobin (HP) were found to be differentially expressed in the serum from patients with NPDR and PDR as compared to healthy controls [10]. The serum levels of α-HS-glycoprotein were increased in PDR patients, whereas the levels of AGP and APOA1 were decreased relative to healthy controls [10]. The downregulation of AGP was not expected, since α1-glycoprotein is involved in pro-inflammatory response [10,107,108]. In a separate study, the inflammatory response protein azurocidin (AZU1) was identified as being elevated in the serum of diabetic patients, especially in patients with diabetic complications including retinopathy [109]. This protein is released by neutrophils in response to inflammatory stimuli on vascular endothelium and is known to play a role in vascular permeability within the retina [110,111].

After identifying many differentially expressed proteins in patient serum and VH, Kim et al. validated a set of twenty-seven biomarkers of mild stage NPDR, including apolipoprotein A-I (APOA1), α-2-macroglobulin (A2M), complement factor H (CFH), and prothrombin (F2), using multiple reaction monitoring (MRM) assays [82]. From these proteins, a four-protein biomarker panel was assembled, including afamin (AFM), apolipoprotein C-III (APOC3), complement factor B (CFB), and kallistatin (SERPINA4), to differentiate between diabetic patients with and without DR with ~85–100% accuracy [82].

### 3.2. Vitreous Humor

The vitreous humor is the gelatinous component of the posterior segment of the eye that gives the eye its spherical shape. In addition to its role in structural support, the transparent nature of the vitreous body aids in light transmission to the retina. Due to the proximal location of the vitreous body to the retina, pathological events in the retina can be monitored through VH examination [4,7,13,16,22,56,101,112,113]. In fact, due to its avascular nature, much of the protein content of the VH comes from the retina itself [81,113,114,115]. VH is often obtained from DR patients during pars plana vitrectomy, in which the entire vitreous body is removed and replaced due to hemorrhage [2,5,22]. Vitreous can also be obtained by a needle biopsy, which is less invasive and can be conducted in the clinic rather than the operating room [22]. VH is obtained from individuals undergoing treatment for a pathological state such as proliferative vitreoretinopathy, rhegmatogenous retinal detachment, idiopathic macular hole (IMH), or epiretinal membrane (ERM) [4,22,63,81,116,117]. In addition, post-mortem VH is obtained from organ donors.

Several proteins in the VH have been identified as biomarkers for different stages of DR. Components of the acute phase response (e.g., α-1-antitrypsin, α-1-glycoproteins, interleukins), complement system (e.g., C3), coagulation pathway (e.g., fibrinogen, prothrombin), and other inflammatory pathways (e.g., VEGF, amyloid-β A4 protein, kininogen-1, metalloproteinase inhibitor 1) have been identified by multiple studies in DR [3,4,11,13,15,39,56,62,63,66,70,71,72,118]. Interleukins have been well characterized for their role in promoting inflammation in eyes with DR [66,119,120,121,122,123,124,125]. Additionally, many members of the apolipoprotein family have been identified in the VH [4,39,63,70,126]. A negative regulator of inflammatory processes, pigment epithelium-derived factor (PEDF) has lower levels in VH from DR subjects as compared to subjects without DR, suggesting that there is not only an increase in pro-inflammatory cytokines, but also a decrease in balancing anti-inflammatory proteins [20,62,63]. Similarly, clusterin (CLU), a protein involved in the regulation of the complement cascade, also had higher levels in control samples relative to DR samples [13,20,39,63]. It has been suggested that clusterin functions in an anti-inflammatory protective role of the blood-retinal barrier [13,67]. Kita et al. showed that plasma kallikrein, a central component of the pro-inflammatory kallikrein kinin system, was elevated in the VH from DR patients [3,15,72]. Gao et al. has shown upregulation of angiotensinogen and downregulation of calsyntenin-1 (CLSTN1), interphotoreceptor retinoid-binding protein (IRBP), interphotoreceptor matrix proteoglycan 2 (IMPG2), extracellular superoxide dismutase (SOD3), and neuroserpin (SERPINI1) in the VH of PDR patients [16].

Several studies have also used VH proteomics to examine the effects of anti-VEGF intravitreal injections on disease progression [3,33,35]. Although Loukovaara et al. observed an increased presence of complement, coagulation, and other inflammatory proteins in the VH of DR patients, they were unable to identify a significant effect of the anti-VEGF agent bevacizumab on these proteins [3]. Interestingly, Wei et al. observed an increase in complement factors, coagulation factors, apolipoproteins, and immunoglobulins following intravitreal injection, while photocoagulation was able to reduce levels of the pro-inflammatory protein osteopontin (SPP1) [35]. Zou et al. compared the VH proteomes of DR patients treated with the anti-VEGF agent ranibizumab and found an expected decrease in VEGF levels as well as a decrease in acute inflammatory response, platelet degranulation, and complement activation proteins [33,34].

### 3.3. Aqueous Humor

Aqueous humor is the fluid in the anterior chamber of the eye; it is produced by the ciliary body epithelium [127,128]. AH is an integral component in many ocular health functions, including nutrient and oxygen supply, removal of metabolic waste, ocular immunity, and ocular shape and refraction [129]. The major constituents of AH are proteins, water, and electrolytes. Although proteins in AH are present in relatively low concentrations compared to blood serum, they are vital in the maintenance of anterior segment homeostasis [65,128,129,130,131,132,133,134,135,136]. Previous studies [130,131,132,133,137], including one of our own [138], have shown significant alteration in several proteins in the AH obtained from glaucomatous eyes. Proteins in the systemic circulation may cross through fenestrated capillaries into the ciliary body, the production center of AH [13,65,139].

In a recent study examining the AH proteomic profile of PDR patients, LC-MS/MS analysis identified 10 proteins associated with PDR [13]. These proteins were involved in a number of biological processes including inflammation and included apolipoprotein A-I (APOA1), apolipoprotein A-II (APOA2), apolipoprotein A-IV (APOA4), and α-1-acid glycoprotein 1 (ORM1) [13]. This finding evidenced a retinal inflammatory response in individuals with PDR in which pro-inflammatory cytokines entered the AH via either the vasculature or the VH [13]. Another proteomic analysis compared the AH of patients with DR to that of patients without diabetes undergoing cataract surgery [65]. Following 2D-DIGE and MALDI-TOF MS, this study identified eleven differentially expressed proteins, three of which are associated with inflammation: apolipoprotein A-I (APOA1), selenoprotein P (SELENOP), and cystathionine β-synthase (CBS). Selenoprotein P plays an important role in maintaining oxidative balance, and was strongly downregulated in the AH of DR patients [140]. Cystathione β-synthase was highly upregulated in DR patients; this enzyme is responsible for the synthesis of hydrogen sulfide and is linked to inflammation and cellular apoptosis [141]. Chiang et al. conducted a study focusing on the proteomic differences of AH from diabetic patients with and without DR [65]. Inflammation-related protein, apolipoprotein A-I, and a number of other proteins related to angiogenesis, structural remodeling, and oxidative stress were identified [65]. Similar to the findings in VH, overall protein concentrations were higher in the AH from DR patients as compared to patients without DR [65].

### 3.4. Tears

Tears are an aqueous solution of proteins, lipids, and other components, and their proteome has been shown to respond to insults such as cataract surgery [17,68,142,143,144]. Tear sampling can provide pathological information in retinal disease and is the least invasive method of biofluid collection, offering a potential method for diagnosis and pre-screening [17,68,69]. The tear proteome of DR patients has become the special focus of several recent studies since the first study by Herber et al. in 2000 [145].

Csosz et al. created a methodology for identifying DR biomarkers using MS analysis of pooled tear samples from healthy controls and diabetic patients at various stages of retinopathy (no DR, NPDR, and PDR) [17,104]. A general decrease in protein content was observed with DR onset, perhaps resulting from defective tear formation or more diluted tears [17]. Furthermore, several proteins were identified as being differentially expressed, including the inflammation-related protein lactotransferrin (LTF) which was significantly upregulated in DR [17]. Lactrotransferrin, along with the other five candidate biomarkers identified by this study (lipocalin 1 (LCN1), lacritin (LACRT), lysozyme C (LYZ), lipophilin A (SCGB1D1), and immunoglobulin λ chain (IGLC1)), were used to develop a machine learning model for diagnosis based on both the combined expression of these and other proteins and clinical images of the retina [17,68,69,104]. In a separate study, β-2-microglobulin (B2M) evidenced a decreased expression in DR patients relative to both healthy controls and diabetic patients without DR [104,146]. In addition to proteomic analysis, tear samples have also been used to examine glycomic differences between DR patients and healthy controls [59,104].

## 4. Post-Translational Modifications as Biomarkers of Diabetic Retinopathy

High mass accuracy instruments have enabled the identification of post-translational modifications (PTMs) in proteins [147,148]. Recent studies have examined the role of protein acetylation and glycosylation in DR pathogenesis [58,59]. MS proteomic approaches have shown that hyperglycemia induces histone acetylation in diabetic rat retinas, which correlates with increased retinal expression of the pro-inflammatory proteins ICAM-1, iNOS, and VEGF [58]. These changes in both histone acetylation and pro-inflammatory cytokine expression were significantly inhibited by a drug used in DR treatment (minocycline) as well as by a histone acetyltransferase inhibitor (garcinol) and histone deacetylase agonists (theophylline and resveratrol) [58]. The correlation between histone acetylation and the expression of pro-inflammatory cytokines suggests that such PTMs and epigenetic regulation may play a role in DR development and progression.

In another study, the inflammation-related proteins antithrombin III (ATIII), clusterin (CLU/APOJ), osteopontin (OPN), and vitronectin (VTN) were found to be present in truncated forms as indicated by unexpectedly low molecular weights obtained via SDS-PAGE/LC-MS/MS. Similarly, a glycomic study on tear proteins found that one O-linked glycan and five N-linked glycans evidenced differences between DR patients and healthy controls [59]. The specific proteins that were glycosylated by these polysaccharides, however, were not identified by the study [59,104].

## 5. Limitations

The primary limitations of large-scale proteomic analysis of VH and AH is the difficulty in obtaining adequate numbers and volumes of samples, as well as the lack of true controls [63]. Obtaining vitreous and aqueous humor samples requires highly invasive procedures that should only be undertaken when necessary for the patient’s health [4,61]. In this way, it is impossible to obtain AH and VH samples from completely healthy living human eyes [4,61]. For these reasons, much recent focus has been turned towards proteomic screening of tear and serum, both of which can be obtained through non-invasive or minimally invasive procedures.

Different pathological states may cause changes in the total protein content, as retinal vascular leakage is known to increase the amount of proteins present in the VH and the AH by introducing non-native proteins [2,4,5,22,39,57,65,81,149]. Furthermore, highly abundant proteins like serum albumin, hemoglobin, and crystallins can mask the detection of lower abundance proteins [3,16,20,22,57,63,70,149]. Depletion of the most abundant proteins using column chromatography can ameliorate some of these issues and enrich proteins that are less abundant.

## 6. Future Directions

Proteomic studies examining the ocular biofluids of diabetic patients and diabetic animal models have made an important contribution to the advancement in our understanding of DR pathogenesis. As high-throughput protein analysis techniques have continued to improve, these studies have great future potential for both biomarker discovery and identification of novel therapeutic targets. It is now becoming possible to accurately characterize the proteomes of very small biological samples, making the use of AH and VH from both patients and diabetic animal models more feasible. Greater dynamic range makes it easier to reliably detect very low abundance proteins, which is particularly important for the use of serum samples in the study of DR [150]. Technological improvements have also expanded our ability to detect post-translational modifications of proteins, making it possible to evaluate differences in glycosylation, phosphorylation, and acetylation states, providing more functional context to proteomic differences between healthy and disease states [151].

Lastly, it is important that these wide-scale proteomic analyses are not limited to the identification of diagnostic biomarkers and therapeutic targets. Proteomic and other “–omic” analyses are also valuable tools to screen the effects of novel therapeutic agents. For example, phlorizin, a naturally occurring compound found in fruit trees and known to have anti-inflammatory and antioxidant effects, was used to treat C57BLKS/J db/db mice [9,152,153]. An iTRAQ proteomic analysis of retinal tissue showed changes in the expression of proteins involved in inflammation, apoptosis, and oxidative stress, which corresponded to functionally validated decreases in retinal cell apoptosis and injury [9]. In conclusion, high-throughput proteomic analyses offer a promising future for the discovery of diagnostic, prognostic, and theraputic monitoring biomarkers, as well as for the development of new therapies for this sight-threatening disease.

## Figures and Tables

**Table 1:** Biomarkers of retinal inflammation in diabetic retinopathy (DR) patients detected using proteomic approaches.

Biomarker	Symbol	Fluid	Comparison	Detection Method	Ref.
**α-1-acid glycoprotein**	AGP	Serum; Plasma; VH; AH	DR/noDR; PDR/Healthy; PDR/NDM; PDR/Post-mortem	2D-DIGE/MALDI-TOF-TOF MS; SDS-PAGE/LC-MS/MS; LC-MS/MS; MRM	[4,10,13,16,82]
**α-1-antichymotrypsin**	SERPINA3	Plasma;VH	DR/noDR; PDR/NDM; PDR/Post-mortem	SDS-PAGE/LC-MS/MS; LC-MS/MS; MRM	[4,16,82]
**α-1-antitrypsin**	SERPINA1	Plasma; VH	DR/noDR; NPDR/NDM; PDR/NDM; PDR/Post-mortem	2D-DIGE/MALDI-TOF-TOF MS; SDS-PAGE/LC-MS/MS; LC-MS/MS; MRM	[4,16,63,82]
**α-2-antiplasmin**	SERPINF2	Plasma;VH	DME/NDM; NPDR/NDM; PDR/NDM	LC-MS/MS; ELISA	[15,83]
**α-2-HS-glycoprotein**	AHSG	VH	DME/NDM; PDR/NDM	SDS-PAGE/LC-MS/MS; LC-MS/MS	[15,16]
**α-2-macroglobulin**	A2M	Plasma; VH	DR/noDR; PDR/Post-mortem	LC-MS/MS; MRM	[4,82]
**Amyloid β A4 protein**	APP	VH	PDR/NDM; PDR/noDR	SDS-PAGE/LC-MS/MS; LC-MS/MS	[13,16]
**Angiotensinogen**	AGT	VH	PDR/NDM; PDR/noDR	SDS-PAGE/LC-MS/MS	[16]
**Antithrombin III**	SERPINC1	VH	PDR/NDM; PDR/Post-mortem	SDS-PAGE/LC-MS/MS; LC-MS/MS	[4,16]
**Apolipoprotein A-I**	APOA1	Plasma; VH; AH	DME/NDM; DR/noDR; NPDR/NDM; PDR/NDM; PDR/Post-mortem	2D-DIGE/MALDI-TOF-TOF MS; SDS-PAGE/LC-MS/MS; LC-MS/MS; MRM	[4,13,15,16,63,65,82]
**Apolipoprotein A-II**	APOA2	Plasma; VH	DME/NDM; DR/noDR; PDR/NDM	SDS-PAGE/LC-MS/MS; LC-MS/MS; MRM	[13,15,16,82]
**Apolipoprotein A-IV**	APOA4	Plasma; VH; AH	DR/noDR; NPDR/NDM; PDR/NDM; PDR/Post-mortem	2D-DIGE/MALDI-TOF-TOF MS LC-MS/MS; MRM	[4,13,63,82]
**Apolipoprotein C-I**	APOC1	Plasma; VH	DR/noDR;PDR/NDM; Anti-VEGF treated PDR/untreated PDR	LC-MS/MS; MRM	[33,82]
**Apolipoprotein C-III**	APOC3	VH	PDR/NDM	SDS-PAGE/LC-MS/MS	[16]
**Apolipoprotein E**	APOE	VH	DME/NDM; PDR/Post-mortem	LC-MS/MS	[4,70]
**ATP-binding cassette subfamily F member 1**	ABCF1	VH	DME/NDM	LC-MS/MS	[70]
**Basement membrane-specific heparan sulfate proteoglycan core protein**	HSPG2	VH	PDR/NDM	SDS-PAGE/LC-MS/MS	[16]
**β-2-microglobulin**	B2M	VH	PDR/NDM	SDS-PAGE/LC-MS/MS	[16]
**β-crystallin A3**	CRYBA1	VH	PDR/Post-mortem	2D-DIGE/MALDI-TOF-TOF MS/MS; LC-MS/MS	[4,20]
**C-reactive protein**	CRP	Serum; Plasma	DR/NDM; DR/noDR; NPDR/PDR	Multiplex Bead Array; ELISA	[74,84,85]
**C-C motif chemokine 5**	CCL13	Serum	Severe DR/Mild DR	ELISA	[86]
**Chitinase-3-like protein 1**	CHI3L1	VH	PDR/NDM	SDS-PAGE/LC-MS/MS	[16]
**Clusterin**	CLU	VH	DME/NDM; PDR/NDM; PDR/Post-mortem	2D-DIGE/MALDI-TOF-TOF MS/MS; 2D-DIGE/MALDI-TOF-TOF MS; LC-MS/MS	[4,20,39,64]
**Cofilin-1**	CFL1	VH	PDR/Post-mortem	LC-MS/MS	[4]
**Complement C1**	C1	VH	DME/NDM; PDR/NDM	2D-DIGE/MALDI-TOF-TOF MS; LC-MS/MS	[39,70]
**Complement C3**	C3	Plasma;VH	DME/NDM; DR/noDR NPDR/NDM; PDR/NDM; PDR/Post-mortem	2D-DIGE/MALDI-TOF-TOF MS; SDS-PAGE/LC-MS/MS; LC-MS/MS; MRM	[4,15,16,63,82]
**Complement C4**	C4	VH	DME/NDM; PDR/NDM; PDR/Post-mortem	2D-DIGE/MALDI-TOF-TOF MS; LC-MS/MS	[4,39]
**Complement C7**	C7	VH	DME/NDM	LC-MS/MS	[15]
**Complement C8**	C8	VH	DME/NDM	LC-MS/MS	[15]
**Complement factor B**	CFB	Plasma	DR/noDR	MRM	[82]
**Complement factor H**	CFH	Plasma; VH	DME/NDM; DR/noDR	LC-MS/MS; MRM	[15,82]
**Complement factor I**	CFI	VH	PDR/NDM; PDR/Post-mortem	2D-DIGE/MALDI-TOF-TOF MS/MS; SDS-PAGE/LC-MS/MS; LC-MS/MS	[13,16,20]
**Estrogen receptor**	ESR1	VH	PDR/Post-mortem	LC-MS/MS	[4]
**Fibrinogen**	FGA, FGB, FGG	VH	DME/NDM; NPDR/NDM; PDR/NDM; PDR/Post-mortem; Anti-VEGF treated PDR/untreated PDR	2D-DIGE/MALDI-TOF-TOF MS; LC-MS/MS	[4,35,39,63]
**Fibronectin**	FN1	VH	Anti-VEGF treated PDR/untreated PDR	LC-MS/MS	[35]
**Gelsolin**	GSN	VH	DME/PDR; PDR/NDM	2D-DIGE/MALDI-TOF-TOF MS	[39]
**Guanylate-binding protein3**	GBP3	VH	PDR/Post-mortem	LC-MS/MS	[4]
**Haptoglobin**	HP	Serum; Plasma; VH	DME/NDM; DR/NDM; PDR/Healthy	2D-DIGE/MALDI-TOF-TOF MS; LC-MS/MS; MRM	[10,15,73]
**Immunoglobulin α chain**	IGHA1	VH	PDR/Post-mortem	2D-DIGE/MALDI-TOF-TOF MS/MS	[20]
**Immunoglobulin γ chain**	IGHG1	VH	PDR/NDM; PDR/Post-mortem	SDS-PAGE/LC-MS/MS; LC-MS/MS	[4,16]
**Immunogloulin heavy chain V-III region BRO**	IGHV3-13	VH	PDR/Post-mortem	2D-DIGE/MALDI-TOF-TOF MS/MS	[20]
**Immunoglobulin κ chain**	IGK	VH	PDR/NDM; PDR/Post-mortem	SDS-PAGE/LC-MS/MS; LC-MS/MS	[4,16]
**Immunoglobulin λ chain**	IGH	VH	PDR/Post-mortem	2D-DIGE/MALDI-TOF-TOF MS/MS	[20]
**Inter-α-trypsin inhibitor heavy chain family, member 4**	ITIH4	VH	DME/NDM	LC-MS/MS	[15]
**Intercellular adhesion molecule 1**	ICAM1	Serum	NPDR/NDM; NPDR/noDR; NPDR/PDR; PDR/NDM; PDR/noDR	Multiplex Bead Array; ELISA	[74,87]
**Interferon γ-induced protein 10**	CXCL10	VH	DME/NDM	Multiplex Bead Array	[66]
**Interleukin-1**	IL1	VH	DME/NDM	Multiplex Bead Array	[66]
**Interleukin-1 receptor antagonist**	IL1RN	VH	DME/NDM; PDR/NDM	Multiplex Bead Array	[66]
**Interleukin-2 receptor**	IL2R	Serum	NPDR/NDM; PDR/NPDR/noDR; NPDR/PDR; PDR/NDM; PDR/noDR	Chemiluminescent Immunometric Assay	[88]
**Interleukin-6**	IL6	Plasma; VH	DME/NDM; nPDR/NDM; PDR/NDM	Multiplex Bead Array; ELISA	[66,89]
**Interleukin-6 receptor**	IL6R	Serum	DR/noDR	Multiplex Bead Array	[74]
**Interleukin-8**	CXCL8	Serum; VH	DME/NDM; NPDR/NDM; nPDR/noDR; NPDR/PDR; PDR/NDM; PDR/noDR	Multiplex Bead Array; Chemiluminescent Immunometric Assay	[66,88]
**Interleukin-10**	IL10	VH	PDR/NDM	Multiplex Bead Array	[66]
**Interleukin-12**	IL12	VH	PDR/NDM	Multiplex Bead Array	[66]
**Interleukin-13**	IL13	VH	PDR/NDM	Multiplex Bead Array	[66]
**Keratin, type II cytoskeletal I**	KRT1	VH	PDR/NDM; Anti-VEGF treated PDR/untreated PDR	LC-MS/MS	[33]
**Kininogen 1**	KNG1	VH	DME/NDM; PDR/NDM	LC-MS/MS	[13,15]
**Lactotransferrin**	LTF	Tear	PDR/noDR	LC-MS/MS	[17]
**Leukocyte platelet-activating factor receptor**	PTAFR	VH	PDR/Post-mortem	LC-MS/MS	[4]
**Macrophage inflammatory protein 1**	CCL3, CCL4	VH	PDR/NDM	Multiplex Bead Array	[66]
**Metalloproteinase inhibitor 2**	TIMP2	VH	PDR/NDM; Anti-VEGF treated PDR/untreated PDR	LC-MS/MS	[33]
**Monocyte chemoattractant protein-1**	CCL2	VH	PDR/NDM	Multiplex Bead Array	[66]
**Monocyte differentiation antigen CD14**	CD14	VH	PDR/NDM	SDS-PAGE/LC-MS/MS	[16]
**Nuclear receptor subfamily 1D2**	NR1D2	VH	DME/NDM	LC-MS/MS	[70]
**Osteopontin**	SPP1	VH	Post-photocoagulation/Pre- photocoagulation	LC-MS/MS	[35]
**Pentraxin-related protein 3**	PTX3	Plasma	DR/NDM; DR/noDR	ELISA	[85]
**Peptidyl-prolyl cis-trans isomerase a**	PPIA	VH	PDR/Post-mortem	LC-MS/MS	[4]
**Peroxiredoxin 2**	PRDX2	Plasma	DR/NDM	MRM	[73]
**Plasma protease C1 inhibitor**	SERPING1	Plasma; VH	DR/noDR; PDR/Post-mortem	LC-MS/MS; MRM	[4,82]
**Plasma serine protease inhibitor**	SERPINA5	VH	PDR/NDM; Anti-VEGF treated PDR/untreated PDR	LC-MS/MS	[33]
**Plasminogen activator inhibitor 1**	SERPINE1	Serum	Dr/noDR	Protein Array	[90]
**Pigment epithelium-derived factor**	PEDF	Plasma; VH	DR/NDM; DR/noDR; PDR/NDM; PDR/Post-mortem	2D-DIGE/MALDI-TOF-TOF MS/MS; SDS-PAGE/LC-MS/MS; LC-MS/MS; MRM; ELISA	[4,16,82,91]
**Protein Dj-1**	PARK7	VH	PDR/Post-mortem	LC-MS/MS	[4]
**Protein FAM3C**	FAM3C	VH	DME/NDM	LC-MS/MS	[70]
**Prothrombin**	F2	Plasma; VH	DME/NDM; DR/noDR; PDR/NDM; PDR/Post-mortem	SDS-PAGE/LC-MS/MS; LC-MS/MS; MRM	[4,13,16,82]
**Retinoic acid receptor responder 2**	RARRES2	VH	DME/NDM	LC-MS/MS	[15]
**Serum amyloid A protein**	SAA	Serum;VH	DME/NDM; DR/noDR	LC-MS/MS; Multiplex Bead Array	[15,74]
**E-selectin**	SELE	Serum	DR/noDR	Protein Array	[90]
**Soluble glycoprotein 130**	sgp130	Serum	DR/noDR	Multiplex Bead Array	[74]
**Stromal cell-derived factor 1α**	CXCL12	Serum	Severe DR/Mild DR	ELISA	[86]
**Transthyretin**	TTR	VH	DME/NDM; DME/PDR	2D-DIGE/MALDI-TOF-TOF MS	[39]
**Tumor necrosis factor α**	TNF	Serum	NPDR/NDM; NPDR/noDR; NPDR/PDR; PDR/NDM; PDR/noDR	Chemiluminescent Immunometric Assay	[88]
**Tumor necrosis factor receptor**	TNFR	Serum	DR/noDR	Multiplex Bead Array	[74,88]
**Vascular cell adhesion protein 1**	VCAM1	Serum	NPDR/NDM; NPDR/noDR; NPDR/PDR; PDR/NDM; PDR/noDR	Multiplex Bead Array; ELISA	[74,87]
**Vascular endothelial growth factor**	VEGF	Serum; Plasma	NPDR/NDM; NPDR/noDR; PDR/NDM; PDR/noDR; NPDR/PDR	ELISA; Multiplex Bead Array	[87,92,93,94]
**Vascular endothelial growth factor receptor 1**	FLT1	VH	Anti-VEGF treated PDR/untreated PDR	LC-MS/MS	[35]
**Vitronectin**	VTN	Plasma; VH	DME/NDM; DR/noDR; PDR/NDM	LC-MS/MS; MRM	[13,15,82]

AH: Aqueous humor; VH: Vitreous humor; DR: Diabetic retinopathy; noDR: Diabetic controls without diabetic retinopathy; PDR: Proliferative diabetic retinopathy; NPDR: Non-proliferative diabetic retinopathy; DME: Diabetic macular edema; NDM: Non-diabetic controls; LC-MS/MS: Liquid chromatography tandem mass spectrometry; SDS-PAGE/LC-MS/MS: Sodium dodecyl sulfate polyacrylamide gel electrophoresis coupled with liquid chromatography mass spectrometry; 2D-DIGE/MALDI-TOF-TOF MS: Two-dimensional gel electrophoresis coupled with matrix-assisted laser desorption/ionization time of flight tandem mass spectrometry; MRM: Multiple reaction monitoring; ELISA: Enzyme-linked immunosorbent assay.
